# Towards a “trauma-informed spaces of care” model: The example of services for homeless substance users

**DOI:** 10.1177/03091325241269757

**Published:** 2024-08-22

**Authors:** Hannah Brais, Mylene Riva

**Affiliations:** 5620 McGill University, Canada

**Keywords:** Homelessness, trauma, spaces of care, substance use, addiction, housing

## Abstract

While clinical practitioners have long recognized the importance of trauma-informed models of care, geographies of care scholars have been slow to engage with and address trauma in its methodologies for better understanding environments that support, or hinder, care for people. Marrying the conceptual contributions of geographies of care, trauma geographies, and geographies of addiction, this paper aims to advance the inquiry of trauma-informed spaces of care. Drawing on the example of the homeless substance user, we present a novel theoretical imperative for considering trauma on both an individual and collective level for advancing spatial interventions for healing in spaces of care.

## I Introduction

Inebriated and tired, the homeless substance user tries to find refuge in the closest emergency shelter. There, they are scrutinized for their substance use and maybe they are refused entry. If they are accepted into the shelter, they are placed in a room with dozens of strangers, vulnerable to theft or attack and likely to have to repeat the process of accessing a shelter the next day. In many jurisdictions, homeless substance users are overrepresented amongst homelessness counts and studies (e.g., Gaetz et al., 2016; Hwang, 2001; North et al., 2010). Despite this, many homelessness resources remain prohibitive to this group, denying them adequate care and housing resources—as illustrated in the all-too-frequent situation described above.

Homeless substance users navigate both environments that cause them harm and environments intended to care for them; these spaces are not mutually exclusive (Dej, 2020; Ecker et al., 2021; Kerman et al., 2023; Willse, 2015). The very nature of being homeless, for many who are unhoused, is to not have access to the needs or privacy of “home” whether they be practical, emotional, or spiritual and having to fulfill these needs in different, often public spaces (Proudfoot, 2017). In absence of the adequate prevention of a homelessness episode, or programming options to be quickly rehoused, many individuals access emergency housing resources, such as a homeless shelter, to avoid the harms of living rough or to try and permanently exit homelessness. While homeless spaces of care can provide essential services for supporting unhoused individuals, these spaces can also be traumatizing (Ecker et al., 2021; Kerman et al., 2023), put someone at-risk of being criminalized (Gowan, 2010), in many ways perpetuate homelessness through inadequate supports that fail to stably house individuals (Dej, 2020; Willse, 2015), and fundamentally undermine a person’s autonomy (Parsell, 2023). In the case of a substance user trying to access a homeless-specific resource, there are numerous barriers that can prevent access. The barriers to services for, or the active refusal of, homeless substance users become a new (and often recurrent) trauma on the top of the existing trauma associated with being homeless (Evans, 2011). Beyond the trauma inflicted through the environments of homelessness, heavy substance users typically carry significant trauma prior to developing an addiction (Maté, 2008; Proudfoot, 2019). For many, substance use is an act of self-isolation that can be used as a protective factor when dealing with traumatization (Linklater, 2020). From this lens we can understand that for the homeless substance user, spaces of care can be heavily enmeshed with a geography of trauma, whether it be embodied (Adams-Hutcheson, 2017), constantly encountered through their environment (Drozdzewski and Dominey-Howes, 2015), or a result of living through the violence of precarity and austerity (Pain, 2019; Shaw, 2019). The intersection of trauma and the need for spaces of care are particularly poignant for homeless substance users, making it a valuable example for better integrating our spatial understandings of trauma into spaces of care.

As health geographers increasingly engage in the spatiality of trauma, there is a need for refining understandings of trauma in our research practices (Pain, 2021). Since the late 1990s, clinical researchers have greatly advanced understandings of trauma-informed care (Reeves, 2015), while health geographers have not taken up trauma in studies about spaces of care. Bridging this gap, we suggest that health geographers assume the ethical responsibility of engaging with trauma to develop a new approach: trauma-informed spaces of care. This paper provides a novel overview of research that conceptually supports a shift towards trauma-informed health geography. It begins by providing a conceptual exploration of spaces of care and geographies of trauma literature. Demonstrating the necessity of a trauma-informed spaces of care, this paper then explores the literature around geographies of homelessness and addiction. Using the example of the homeless substance user, the role of trauma is made clear as an essential consideration for future spaces of care research, with examples of trauma-informed spaces of care provided.

## II Towards trauma-informed spaces of care

There is a particular urgency for health geographers to make the conceptual turn toward embracing trauma-informed models for geographies of care. As Pain’s (2021, p. 985) call-to-arms highlights, “geographers have been slow to explore trauma-informed care and environments. What would trauma-informed geographies, research and pedagogy look like?”. Notably, as the field responsible for linking the relationship between space, care, and people, scholars examining geographies of care could productively examine trauma in its many spatial forms and how it impacts people’s experience of space, place, and ultimately care. As clinical researchers over the past two decades have adopted a trauma-informed care in developing frontline services (Reeves, 2015), the role of trauma requires a central consideration in our practices as health geographers. In this section we present an overview of both geographies of care and trauma geographies, using the example of the homeless substance user, and propose merging the two subfields to produce a trauma-informed spaces of care model.

### 1 Geographies of care

Geographies of care scholarship engages with the concept of care broadly defined as “the provision of practical or emotional support to those who would otherwise be unable to undertake activities of daily life due to physical or mental disability, illness, injury, or an age-related condition” (Milligan 2014: p1). Beyond this, geographies of care spatializes “the interrelationships between people, places, and care” (Milligan 2014: p1). This framework expands on the scholarship on therapeutic landscapes (Gesler, 1992), which recognizes that care and the specific spaces (as opposed to larger landscapes) it occupies are complex and impact the health and wellbeing of the individuals accessing it. This framework also takes an important look at how policies shape the practices and spaces of care, notably with an emphasis on how all levels of policy ultimately impact the person directly giving or receiving care. For example, early research on spaces of care was interested in the way shifting policies around care for aging adults impacted the where, who, and how of caring for this group (Milligan, 2017). More conventional notions of care (e.g., caring for children or health care) are often the subject of interest for spaces of care scholars. We propose, following a number of other scholars (e.g., Conradson 2003; Evans 2011, 2012; Johnsen et al. 2005; Knowles 2000; Mee 2009; Parr 2019), that housing provision and services for those without housing are an important form of care. Core to this idea is that housing is an essential part of a person’s wellbeing: offering housing is as vital as any other form of care that a person may seek out as it provides both a physical and ontological sense of security (Padgett, 2007).

Within geographies of care, the role of the homeless subject is shifted from a victim and charity recipient to someone who has a powerful relational role and voice in a multi-scalar dynamic of policy, service provider, and service user (Power and Williams, 2020). Returning to the homeless substance user, we see the challenges they experience as directly impacted by this multi-scalar dynamic. For example, in many contexts, they cannot access the emergency shelter while significantly inebriated because they pose too much of a liability to the service provider, or because local policies or funding bodies dictate that service providers cannot accept individuals who are too intoxicated. Under this lens, homeless service providers, operating under specific policies, are seen as spaces of care that create organizational structures and practices and deliver care under these policies (Mee, 2009; Power and Williams, 2020). Thus, this geographies of care approach looks closely at how the organizational space harbors a sense of caring for the individual or not (Conradson, 2003; Milligan, 2003). This framework offers a valuable opportunity for understanding the barriers homeless substance users face with regards to homeless and housing services: it takes the experience of an individual accessing (or trying to access) services, places it in conversation with the models employed by services, and situates those models within the policies that govern them.

### 2 Homeless spaces of care

There is a modest body of scholarship that investigates examples of spaces of care for the homeless. Kearns and Smith (1994) highlighted the value of developing a critical geographical lens for examining a deeper phenomenology of homelessness, notably underlining a spectrum of homelessness through lived experience. This early work helped inform the foundations of geographies of care (Milligan, 2017) and clearly placed the homeless subject, as a recipient of care, in a powerful relational role with their environment. More recent literature offers insights into the relational value of homeless individuals and the spaces upon which they rely (Conradson, 2003; Evans et al., 2015; Johnsen et al., 2005). As these authors often demonstrate, and individuals accessing these services know, the range of homeless spaces of care available is often questionable in their ability to care: they are often traumatogenic, fail to instill dignity, are unsafe, and perpetuate a paternalistic charity model of care (Dej, 2020).

In the last twenty years, geographies of care scholars have taken up the question of homelessness more carefully. Conradson (2003) draws on the specific example of a drop-in center in Bristol, England, to qualify the low-barrier service (typically a space where individuals can enter inebriated with limited rules and restrictions) as a space of care, distinct from a therapeutic landscape, in an urban setting. Notably, Conradson’s emphasis is the relational experience of the end user and how this in itself provides complexity on the nature of care within these spaces. Significantly, he challenges an exclusively negative reading of power relations within this form of space of care and makes the argument that spaces of care can offer an *ontology of hope* by engaging with the different subjectivities of people who use this space and find meaning and positivity in their relationship with it. Evans et al. (2015) also examine a low-barrier homeless service, exploring the range of experiences of individuals receiving care. Evans (2011) then goes further to describe the homeless services as a political feature that provides citizenship and legitimacy to those who access it. He uses the example of a low-barrier service to illustrate how offering care, with no barriers, allows service users to regain a better sense of self as they are offered inclusion where their lives constantly intersect with exclusionary spaces. This point of view further legitimizes the homeless space of care as one that carries a multi-scalar complexity of people, spaces, care, and policies. As Conradson (2003) and Evans’ (2011, 2015) work suggests, spaces of care for homeless individuals, and the structural violence these spaces perpetuate or protect from, are ripe for the integration of trauma-informed care solutions.

On a more macro level, Cloke, May, and Johnsen (Cloke et al., 2011) reconsider the urban spatiality of homelessness through a spaces of care framework. Their work challenges assumptions about homeless spaces to demonstrate the complexity of homeless governance and to allocate value to the end-user experience through their specific feelings. Much like Conradson’s (2003) reflection on an ontology of hope, Cloke et al.’s work is of particular interest because they qualify the intent, and in turn the nature of care, of homeless-serving organizations as a complex dynamic between different levels of power. They describe this dynamic as a balance between conforming to a mandate of control from governments in managing the urban homeless population and genuinely offering an act of care based on altruism or a more religious “agape” (a Christian love for one’s fellow man as an embodiment of one’s love for god).

As the complex nature of spaces of care for homeless individuals demonstrates, the multi-scalar dynamic of people, spaces, and care (Milligan, 2014) as embodied through policy, service provider, and service user (Power and Williams, 2020), offers extensive opportunity for challenges to delivering and receiving care. Within these different scales, challenges manifest in elements like the inadequate upkeep of the homeless space of care, negative perceptions of homeless individuals or people who use drugs or alcohol, or prohibitive policies for creating adequate spaces. These challenges are particularly amplified by the insidious nature of trauma, which manifests itself both spatially and temporally in ways that complicate conventional approaches to care.

### 3 Trauma geographies

All trauma is grounded in space. Structural factors define spaces, and these same structural factors drive a person or group’s traumatization (Lucero et al., 2023). The often run-down spaces that accommodate homeless substance users offer a reminder of this: because these spaces are for people living in poverty whose behavior is considered deviant, they are not worthy of more resources or maintenance. In turn, these spaces of care often take a carceral feel where spaces are designed to allow for easy supervision and forceful intervention, the food or other goods provided are whatever is available freely or cheaply, and the model of intervention generally reflects the sense that it is because of individual failings that people find themselves there, often provided by staff or volunteers with little training (Dej, 2020; Parsell, 2023). The people accessing these spaces have lived through the structural violence of poverty, in of itself traumatogenic; these spaces, also treated as less-than-worthy, reflect this social status of lesser. Many spaces that accommodate homeless individuals embody the very structural trauma that a person has lived through and must reexperience as they try to seek refuge or care. The investigation of space in understanding trauma, and the surrounding contested structural factors of the space, is essential. Engaging with this inquiry, the “spatial turn” of trauma in geography can be seen as an important paradox, as trauma can also completely defy a linear spatial grounding: trauma can be experienced when directly engaging with a space, but it is also the space carried within individuals at the locations of previous traumatic events (Coddington and Micieli-Voutsinas, 2017). This internal trauma can be an individual event or a compounding set of spaces experienced through regular and mundane structural violence. As geographers, we must contend with both the more complex internal traumatic spaces (Coddington and Micieli-Voutsinas, 2017) and the linear traumatizing potential of external spaces (Drozdzewski and Dominey-Howes, 2015). To ultimately consider a trauma-informed space of care, a comprehensive portrait of the different spatial elements tied to trauma is necessary.

Within geography, spatialized studies of substance use have typically been characterized as a geography of addiction, qualifying substance use under the term “addiction.” The term “addiction” is problematic, notably if we are to consider that traumatizing and structural violences both lead someone to a dependence on substances and define spaces that are meant to support them (Proudfoot, 2017). “Addiction” represents a pathology whereby a person’s body has come to be dependent on a substance; this pathology renders invisible the experience of being traumatized and treats addiction as the illness, rather than the symptom of being subject to violence. While we challenge the use of “addiction” employed by other geographers, we also engage with extensive literature that continues to employ this term and the broader field of geographies of addiction and acknowledge that the use of “addiction” is meant as a (critical) engagement and not an endorsement of addiction as a pathology. The following section provides an expanded revision of conventional understandings of addiction in geography.

Spatializing the so-called addiction can suggest a shift from therapeutic landscapes to traumatic landscapes, which implies that the environment navigated by a substance user is one defined in opposition to being therapeutic and thus potentially “addictogenic” (Proudfoot, 2019). In an “addictogenic” space, the substance user is further anchored into a dependence to substance(s). For example, when experiencing trauma that either precedes or accompanies homelessness in an emergency shelter, someone may consume more to deal with traumatic pain. Considering the literature around harm reduction, the question of the “addictogenic” environment may be more aptly referred to as the “risk environment,” while also recognizing it as a spatial point for intervention (or care), rather than simply a space that causes addiction (Rhodes, 2002). Recent scholarship has unpacked the role of trauma theory in geography and is particularly productive for considering homelessness and addiction. As trauma exists in the subject as an anchored part of their past, it evolves to merge with their present and future, obscuring time and place as they live the traumatic event recurringly in different spaces and times (Adams-Hutcheson, 2017); this can be better understood as the “temporal dysphoria” of trauma (Caruth, 2014; Pain, 2019). Temporal dysphoria describes a condition in which the traumatized subject cannot live in the present time and place as they are regularly mentally caught in the time and place of traumatic event(s). The effect of regularly oscillating between the present and past distorts a linear experience of time and place for the subject, hence a temporal dysphoria (Caruth, 2014). Thinking once again to the homeless substance user trying to access shelter, while they are potentially mentally returned to the space and time when they were traumatized (e.g., loss of housing), or the recurrent violence of living in extreme poverty (e.g., regularly being unable to access basic necessities), they are also being exposed to new forms of trauma (e.g., being robbed in an emergency shelter).

From another perspective, trauma also has a contagious value that, among other effects, travels through different spaces as individuals who experience similar traumatic events may connect with the other’s traumatic event or site (Coddington, 2017). Coddington and Micieli-Voutsinas (2017) employ Berlant’s concept of *cruel optimism* (Berlant, 2011) to further illustrate the insidious nature of spatializing trauma: trauma goes beyond a traumatic event and is rooted an unfounded hope or *optimism* for something better, a promised relief. The unsatisfied hope for something promised, or something better, becomes the trauma and is embodied in the person living it. This concept of *cruel optimism* can be productively extended to those seeking a space of care who are experiencing homelessness and using drugs or alcohol. The urban environment incessantly suggests, through its abundance of options, that they should access resources to exit homelessness but the barriers in place to these resources (often in the form of abstinence) make it difficult or impossible to do so. Under this reading, a trauma-informed spaces of care challenges the value of care in these settings: substance use is often a symptom of trauma and the forms of violence that create trauma, thus refusing entry because of abstinence-based policies are perpetuating a traumatizing *cruel optimism* where the individual is sent back into traumatogenic environments that drive their substance use, while further denying them care. Another reading of geographies of trauma suggests something far more literal, in that the space of trauma is located simply in the space where the traumatic event took place (Drozdzewski and Dominey-Howes, 2015). Practically, this is also important, as research suggests homeless substance users will often avoid certain resources (spaces of care) because of adverse experiences at the site itself (Krusi et al., 2010; Miewald et al., 2018).

Between a much more literal interpretation of traumatic space (Drozdzewski and Dominey-Howes, 2015) and an embodied form of traumatic space (Adams-Hutcheson, 2017; Coddington, 2017; Coddington and Micieli-Voutsinas, 2017), emerges another understanding to frame the experience of the homeless substance user: that of chronic urban trauma (Pain, 2019). Chronic urban trauma draws directly from the concept of chronic or complex trauma (Herman, 1992) and the idea that, rather than in a punctual and isolated traumatizing moment (typical of a classic post-traumatic stress disorder diagnosis) (American Psychiatric Association, 2013), trauma can be based in recurrent and often mundane ways (such as domestic violence) as experienced through the violence of the city (albeit with clear implications for non-urban environments). Spatializing chronic trauma, Pain (2019) describes a *slow violence* experienced through the urban environment recurringly through a taken-for-granted, built-in traumatic stress. This trauma is both constantly perpetuated in the built environment and embodied in the traumatized subject, who both suffers under, and resists against, overwhelming structural forces, such as poverty, racism, colonialism, ableism, homophobia, transphobia, or misogyny, that perpetuate their condition (Pain, 2021; Tyner, 2020). From this, it is easy to understand a homeless substance user as a subject of chronic urban trauma: they carry whatever trauma may accompany or drive their substance use (Maté, 2008; Proudfoot, 2019) and are (re)traumatized in regular and mundane ways by their environment. The urban environment reminds the homeless substance user that they are lesser because of their lack of stable housing or substance use. Elements like the spaces of care that remind them of being lesser through their inadequacy or unavailability, being pursued by the police for carrying out “private” activities in public spaces, or the constant disdain of strangers compound into a chronic form of traumatic stress. This stress is not marked by a single instance but by a common thread of small, regular, and violent acts against a person’s wellbeing.

Building on health geographers’ reflections on the nature of traumatic spaces, we posit that we have a responsibility beyond observing the relationship between trauma and space, which is to consider the role of space in healing those who occupy it. Following in the conceptual tradition of therapeutic landscapes (Gesler, 1992) where the relational value of how spaces and humans interact can define how spaces offer wellness to humans, the relational factor of spaces and trauma needs to be considered. This relational factor can benefit from Herman’s (2023) (albeit not a geographer but a clinician) more recent definition of trauma: a collective problem where the “strong” impose their will, the “weak” submit, bystanders seem helpless/willfully blind/indifferent/complicit, and there is no justice for the “weak.” As Herman’s argues, the greatest violence imposed on the victims of trauma is typically not the initial act of imposition but the ambivalence or weakness of bystanders. By bystanders, Herman includes institutional or structural bystanders; considering this through a geographical lens, spaces of care can easily embody the passivity of an institutional bystander in this dynamic of trauma. Applying Herman’s reading of institutions that perpetuate violence against women, to the example of homeless spaces of care (such as the homeless shelter) we find an example of an institution and space that has historically been front-row to the devastation of the structural factors that lead someone to homelessness (poverty, racism, colonialism, as some common examples) and has consistently failed to address these structural causes. Within this passivity comes the bystander status of spaces (of care). According to Herman, if the subjects of trauma are to heal, the role of the bystander is to first and foremost acknowledge the harm done and provide a collective vindication. She proposes a “moral community” for this healing where the collective understands that “harm to one is harm to all”; to apply this to the context of a homeless space of care this could look like a recognition on the part of institutions and active advocacy for the eradication of poverty and a right to housing. Considering the relational value of humans and spaces for healing, in this example, the space can recognize the harm done and offer an opportunity for creating a “moral community” that now protects and heals the subject(s) of trauma. Going forward for considering a trauma-informed spaces of care, above all, an understanding of spaces as bystanders in trauma helps center interventions for healing.

### 4 The necessity of a trauma-informed spaces of care

Recent years have seen a radical increase in the prevalence and commonplace use of trauma-informed models of care (TIC), albeit with often poorly defined application (Becker-Blease, 2017). Beyond the frequently nebulous design and application of trauma-informed care, criticisms of trauma-informed care models are often centered around the lack of evidence in their success (Ardino, 2014). As trauma is often under-diagnosed and under-recognized, much as a result of a slow societal recognition of the effect of adverse life events, trauma-informed practices are reciprocally difficult to measure in their effectiveness as the people they benefit often likely fall under the radar of formal diagnosis (Bransford and Cole, 2019). TIC is also critiqued for overlooking staff experiences of trauma (Olivet et al., 2010), as well as for being difficult to implement in settings where clinicians are reticent to acknowledge the role of trauma or change their practices (Bateman et al., 2013), or where service providers may not have access to the resources necessary to adequately and regularly train staff (Bransford and Cole, 2019; Kramer et al., 2015). Regardless of its limitations, trauma-informed practices provide an important opportunity to consciously engage with the realities of individuals living with trauma and to provide better care accordingly.

Within clinical contexts, where the majority of research on trauma-informed practices has occurred (Reeves, 2015), the emphasis is typically on questions of individual pathology (Becker-Blease, 2017) where medical professionals are responsibilized with ensuring that traumatized individuals are able to access appropriate care and are not retraumatized through accessing medical care. This takes the form of establishing trauma screening and patient disclosure, provider–patient relationships, minimizing distress and maximizing autonomy, and multidisciplinary collaboration and referrals (Reeves, 2015). Concretely, this can look like medical staff asking a patient about a difficult childhood inasmuch as it can look like having a clinical setting with limited sensory evidence of other patients (e.g., Liddicoat 2019). This general approach to trauma-informed care places a focus on the individual’s experience through care but does not typically engage with broader concerns of structural-level traumas such as racism, classism, colonialism, misogyny, ableism, homophobia, and transphobia, among many others (Becker-Blease, 2017). Considering individual trauma pathologies, such as post-traumatic stress disorder where individuals are traumatized following a specific event (American Psychiatric Association, 2013) or complex trauma where individuals have been exposed to recurrent trauma (such as domestic violence) (Herman, 1992), in the care design is certainly essential, but it overlooks the structural traumas even possibly informing the individual pathology. As Pain (2021) emphasizes, structural harms can cause the recurrent and mundane chronic urban trauma they take concern with in their earlier work (2019). This echoes the institutional violence flagged by Herman (2023). As such, a trauma-informed care model must also take into consideration these broader structural traumas (Becker-Blease, 2017) and fundamentally focus on fostering a sense of safety for those who use it (Linklater, 2020) with the end goal of healing trauma (Herman, 2023). In practice, this takes form in a commitment on the part of scholars of geographies of care to consider the experiences and ontology of groups that have traditionally been subject to repetitive and regular traumas. Given the broad application of trauma-informed models, and the importance of addressing both individual and group-level structural traumas, the question of trauma-informed spaces of care becomes more important to adequately define and employ.

Geographies of care focuses on a multi-scalar analysis of people, place, and care; applying this through a trauma-informed lens requires merging our spatial understandings of trauma at these scales, while recognizing that they are enmeshed with one another. Considering first of all the *spatial dysphoria* of trauma (Caruth, 2014) there is a necessity to recognize that *people* with trauma do not always engage with the present without carrying the past and the future actively with them. In turn, *spaces* that evoke triggering memories further distort someone’s ability to receive *care*, in that place, in the present tense without fixing them in the traumatic past or anxious future. This is especially true if the space of care is also the site of a traumatic event; people will actively avoid care at this place (Drozdzewski and Dominey-Howes, 2015). Avoiding, or considering, triggering someone’s trauma through environmental interventions is not novel in a clinical setting (Reeves, 2015) but it does add an extra level of responsibility to health geographers critically examining the relationship of people, spaces, and care. Notably, geographers need to consider if a place evokes something likely traumatogenic or is in of itself traumatogenic; the question of adequate care becomes obscured by trauma in both cases.

In another spatial reading of trauma, spaces contain a contagious potential where trauma can travel between individuals and groups of people who have similar or shared experiences, where the location of a traumatic event can be taken up with others who relate to it (Coddington, 2017). Applying this concern for the contagiousness of trauma, Liddicoat (2019) examines spaces of care for self-harmers where she qualifies an “affective spectrality”; that is the negative impact the lingering presence of another patient (such as the indentation in a chair or the smell of cologne) has on another patient. In this reading of traumatic spaces, Coddington’s (2017) concern for contagious trauma is particularly evident for individuals potentially pathologically sensitive to the trauma of others in a space of care. Despite its particularness, Liddicoat is pointing to something that should be of concern within a trauma-informed geographies of care: the effect that other recipients of care have on one another. Taking this a step further, there is an invitation for geographers to consider mitigating the effects of having multiple traumatized people access care in one space and to reflect on the space as a healing agent.

## III Applying trauma-informed spaces of care: The example of the homeless substance user

Applying a trauma-informed model to geographies and spaces of care allows for a more appropriate consideration of the question of services for homeless substance users, while confronting the often inadequate and traumatogenic model of services in practice. To apply this model, a better engagement with the existing geographic understandings of the so-called addiction, and reflections on trauma, is in order. The following section proposes a merging of both geographies of addiction and geographies of trauma’s conceptual frameworks, with a discussion on the relationship between substance use and trauma. Finally, this section offers a conceptual proposal for addressing trauma-informed spaces of care for homeless substance users.

Despite the value of spatializing substance use, the study of geographies of addiction continues to be limited and with distinct conceptual pitfalls (Jayne et al., 2006; Wilton and Moreno, 2012). Geographers have often focused on the addiction itself, or the addict, and how they behave throughout space (Duff, 2012; Jayne et al., 2008; Malins et al., 2006), rather than analyzing how space, and all the associated structural factors of a space, drives addiction. This view inadvertently pathologizes the addiction through centering the analysis on the addiction, rather than the environmental and structural factors that drive it (Proudfoot, 2017). Considering the relationship between addiction and space, we draw on Proudfoot’s (2019) reading of addiction, or substance use, that considers it a certain product of space, rather than simply co-occurring with it. This reading suggests that the link between space and addiction is often trauma, notably that trauma can be perpetuated by space onto the person with an addiction. Under this reading, geographies of addiction merges with geographies of trauma (Proudfoot, 2019). We go further to suggest that this is an inherent consideration for spaces of care to consider how spaces must be conceptualized to heal different forms of trauma.

One challenge associated with considering addiction in geography is a failure to adequately define it: as observed by Proudfoot (2019), addiction has typically been loosely qualified and explored in biomedical terms as a certain sickness of the body, mind, or spirit. Echoing this, DeVerteuil and Wilton’s 2009b review of the field suggests geographers have examined substance addiction through the lenses of (i) consumption of drugs and alcohol and public regulation with an interest in the management and oversight of who can drink and use drugs and where (Kneale and French, 2008); (ii) a public health issue that considers the ways in which the use of drugs and alcohol affect people’s wellbeing while imposing a heavy cost on society; (iii) social geography and identity through examining the ways in which using drugs and alcohol create different relational subjectivities in space. This final addition to the field is of particular interest, as it offers an opportunity to question less the behavior of the addict and consider more profoundly the structural factors that inform it. Notably, a social model of addiction suggests investigating treatment options that respect the agency of the substance user, inviting a spaces of care approach (DeVerteuil and R Wilton, 2009b; DeVerteuil and RD Wilton, 2009a). In the same spirit, Proudfoot (2019) suggests applying a geographical lens to study addiction, that is, one that uses the social model of addiction, to present addiction as the product of structural causes, such as poverty. Notably, he draws on other scholars, outside of geography, such as Maté (2008) and Alexander (2010), who have engaged with this model to present comprehensive portraits of addiction, specifically examining addiction in the space of the Downtown Eastside in Vancouver, British Columbia, Canada. Considering trauma as a wound of structural violence, addiction also serves as one manifestation of self-isolation as a protective factor (Linklater, 2020). This social model suggests that addiction is driven by a mosaic of social, structural, spiritual, and medical factors operating across different spatiotemporal scales that render some individuals far more vulnerable than others to chronic addiction (whether to drugs or alcohol, or other addictive behaviors). Amongst scholarship in and outside of geography, these place-based understandings of addiction underline that its driving factors point to larger structural concerns than the addiction itself.

From a clinical perspective, trauma-informed practices have long been considered essential within homeless services, particularly for individuals with addictions, but with slow uptake (Hopper et al., 2010). Naturally, the most trauma-informed practices are efforts that prevent a person from experiencing homelessness altogether (Shinn and Cohen, 2019), or would quickly rehouse them (Padgett et al., 2016). In absence of an adequate presence of these kinds of interventions, trauma-informed care models become even more central to consider as emergency housing models put service users at a higher risk of (re)traumatization (Kerman et al., 2023). Central to considering trauma-informed models of care for homeless substance users, there remains the application of harm reduction models (Pauly, 2008) typically where individuals can consume alcohol or drugs on-site to minimize the negative effects associated with using substances in an unsheltered or precarious location. Harm reduction can reduce the amount of retraumatizing events that this population faces while using because they are able to use in safer spaces. Barriers to adopting a harm reduction model fit squarely within the concerns of geographies of care; harm reduction is limited by policies, the stigma of people towards alcohol and drug use, and inadequate spaces to accommodate harm-reduction models. Harm reduction is only one self-evident concern that can be taken up by a trauma-informed geographies of care model for homeless substance users: if we understand that being homeless and experiencing addiction typically means navigating a myriad of traumatogenic (as in, can cause trauma but is not inherently traumatic) spaces, then geographies of care can and should take up the concern of care within these spaces while considering the reciprocal complexity this carries for people with trauma and those who care for them (understanding that those two are not mutually exclusive). Another important consideration is the aforementioned role of spaces of care as bystander institutions who have sat passively to the structural traumas of the people who occupy them (Herman, 2023). From this perspective, if spaces of care have a responsibility for healing trauma and not simply just considering it, they also need to evaluate their complicity in individual, collective, and structural traumas. From there they need to acknowledge the harm done by the space and allow the space to become one of moral community, where victims of trauma are supported by a broader community and one another. Particularly as it relates to substance users, addiction often represents a protective factor that allows for self-isolation; spaces of care need to offer communal healing to break the isolation of trauma (Linklater, 2020) while providing a protective moral community (Herman, 2023).

Finally, returning to the homeless substance user and looking forward to a space of care that is less traumatogenic, and thus less addictogenic, we need to consider environments that treat substance use as a response to trauma, and not as a stand-alone pathology. The spaces must also offer collective forms of healing and atonement from institutions that have been complicit in individual and structural harms, even if those institutions are the same spaces meant to care for the homeless substance user. As demonstrated by the literature on traumatic spaces, the investigation into trauma is complex and multi-scalar, while both external and internal to people. Speaking to how spaces of care need to challenge their roles as institutional bystanders to trauma, existing spaces of care must also be agents of advocacy. These spaces have the responsibility to simultaneously recognize that they have perpetuated harm and trauma in their own institution and are actively changing, while also mobilizing larger societal powers to recognize harm done and to improve. Ahead of us is the great challenge of inquiry into the vast spatial nature of trauma, as informed by addiction, how people and care can and should interact with those spaces, and fundamentally the role of space in healing trauma.

## IV Conclusion

Echoing advances in trauma-informed clinical models, there is a conceptual and practical need for geographies of care to adopt a trauma-informed lens. One critical question underlining the urgency for this turn is the case of homeless substance users seeking spaces of care. Understanding the complex and dynamic link between the spatial nature of substance use and trauma, alongside the troubling limitations of spaces of care for homeless individuals, this group signals the necessity of considering trauma in examining the link between people, spaces, and care. In practice, this looks like the careful examination of the role of trauma in the multi-scalar spatial understandings of geographies of care; for example, considering the spatial dysphoria of trauma where individuals carry both traumatic spaces within them and navigate traumatogenic spaces (Caruth, 2014), or considering how structural forms of complex trauma such as racism, misogyny, or homophobia can further taint the experience of accessing or delivering care (Pain, 2021). It additionally calls for geographers to consider how the relational value of people and space can provide healing potential to those living with trauma (Herman, 2023).

Given this complexity and subjectivity of the spatial value of trauma, a trauma-informed space of care for the homeless substance user would necessarily have to begin with the spatial needs of the individual. Simply, in addition to asking the individual, “what do you need to be adequately cared for?” trauma-informed spaces of care need to ask, “where do you need to be cared for?”. Concretely these are flexible models of spaces of care that meet individuals where they need to be, rather than where the care has been placed by default. One example of this is street outreach teams, or mobile health and psychosocial intervention teams, that meet individuals out of conventional homeless spaces of care. If we are considering a more fixed example of a trauma-informed spaces of care for homeless substance users, examples include service providers that offer a range of spaces and services that adapt to the needs to the individual and allow the individual to choose the spaces and services they need, all with a vision of practices that avoid retraumatizing service users. Services like the Victoria Cool Aid Society in Victoria, British Columbia, Canada, have taken this idea by offering a wide range of spaces specifically adapted to the needs of substance users. Such spaces include low-barrier community space where individuals can come and go as they need while accessing basics like food and shelter; a range of safe and comfortable supervised consumption sites that are adapted to specific forms of consumption (i.e., inhalation areas, injection sites with unlimited clean injection material, and designated drinking spaces, all with quick access to overdose support); well-designed and integrated private sleeping accommodations; and on-site mental and physical health staff. Importantly, staff members at the Victoria Cool Aid Society are consistently trained in trauma-informed practices with an emphasis on supporting individuals to becoming stably housed, if that is where they want to be, regardless of whether or not they continue to consume drugs or alcohol (Victoria Cool Aid Society, 2024). This is one example of a space of care that has carefully considered the role of trauma in its spatial design and focused on meeting individuals where they are at.

Within geographies of care, if we are to take up the challenge of honestly confronting the role of trauma in spaces, people, and care, we must apply new ways of understanding the dynamic and mobile nature of trauma. Going forward, we have the obligation to adapt our methodologies for these flexible considerations of trauma. This means going further than acknowledging trauma as less of a pathology but something tangled up in both spaces and the ways in which we care for one another.

## References

[bibr1-03091325241269757] Adams-Hutcheson G (2017) Spatialising skin: pushing the boundaries of trauma geographies. Emotion, Space and Society 24: 105–112.

[bibr2-03091325241269757] Alexander B (2010) The Globalisation of Addiction: A Study in Poverty of the Spirit. Oxford ; New York: Oxford University Press.

[bibr3-03091325241269757] American Psychiatric Association (2013) Diagnostic and Statistical Manual of Mental Disorders: DSM-5. 5th edition. Washington, D.C: American Psychiatric Association.

[bibr4-03091325241269757] Ardino V (2014) Trauma-informed care: is cultural competence a viable solution for efficient policy strategies? Clinical Neuropsychiatry 11(1): 45–51.

[bibr5-03091325241269757] Bateman J Henderson C Kezelman C (2013) Trauma-informed care and practice: towards a cultural shift in policy reform across mental health and human services in Australia. A national strategic direction. Mental Health Coordinating Council 4: 1039856216657698.

[bibr6-03091325241269757] Becker-Blease KA (2017) As the world becomes trauma–informed, work to do. Journal of Trauma & Dissociation 18(2): 131–138. 28145820 10.1080/15299732.2017.1253401

[bibr7-03091325241269757] Berlant L (2011) Cruel Optimism. Durham: Duke University Press.

[bibr8-03091325241269757] Bransford C Cole M (2019) Trauma-informed care in homelessness service settings: challenges and opportunities. In: Homelessness Prevention and Intervention in Social Work: Policies, Programs, and Practices. Berlin: Springer, 255–277.

[bibr9-03091325241269757] Caruth C (2014) Traumatic temporality: an interview with ean laplanche. In: Listening to Trauma: Conversations with Leaders in the Theory and Treatment of Catastrophic Experience. Baltimore: John Hopkins, 25–46.

[bibr10-03091325241269757] Cloke P May J Johnsen S (2011) Swept up Lives? Re-envisioning the Homeless City. Hoboken: John Wiley & Sons.

[bibr11-03091325241269757] Coddington K (2017) Contagious trauma: reframing the spatial mobility of trauma within advocacy work. Emotion, Space and Society 24: 66–73.

[bibr12-03091325241269757] Coddington K Micieli-Voutsinas J (2017) On trauma, geography, and mobility: towards geographies of trauma. Emotion, space and society 24: 52–56.

[bibr13-03091325241269757] Conradson D (2003) Spaces of care in the city: the place of a community drop-in centre. Social & Cultural Geography 4(4): 507–525.

[bibr14-03091325241269757] Dej E (2020) A Complex Exile: Homelessness and Social Exclusion in Canada. Vancouver: UBC Press.

[bibr15-03091325241269757] DeVerteuil G Wilton R (2009a) Spaces of abeyance, care and survival: the addiction treatment system as a site of `regulatory richness. In: Political Geography. The Boulevard, Langford Lane, Kidlington, Oxford Ox5 1gb. Oxon, England: Elsevier Sci Ltd.

[bibr16-03091325241269757] DeVerteuil G Wilton RD (2009b) The geographies of intoxicants: from production and consumption to regulation, treatment and prevention: the geographies of intoxicants. Geography Compass 3(1): 478–494.

[bibr17-03091325241269757] Drozdzewski D Dominey-Howes D (2015) Research and trauma: understanding the impact of traumatic content and places on the researcher. Emotion, Space and Society 17: 17–21.

[bibr18-03091325241269757] Duff C (2012) Accounting for context: exploring the role of objects and spaces in the consumption of alcohol and other drugs. Social & Cultural Geography 13(2): 145–159.

[bibr19-03091325241269757] Ecker J Aubry T Sylvestre J (2021) Experiences of LGBTQ adults who have accessed emergency shelters in a large urban city in Canada. Social Work in Public Health 37: 1–18. 34542018 10.1080/19371918.2021.1976345

[bibr20-03091325241269757] Evans J (2011) Exploring the (bio)political dimensions of voluntarism and care in the city: the case of a ‘low barrier’ emergency shelter. Health & Place 17(1): 24–32. 20605512 10.1016/j.healthplace.2010.05.001

[bibr21-03091325241269757] Evans J (2012) Supportive measures, enabling restraint: governing homeless `street drinkers. In: Hamilton, Canada. Social & Cultural Geography. 2-4 Park Square, Milton Park, Abingdon Ox14 4rn. Oxon, England: Taylor & Francis Ltd.

[bibr22-03091325241269757] Evans J Semogas D Smalley JG , et al. (2015) “This place has given me a reason to care‘”: understanding ‘managed alcohol programs’ as enabling places in Canada. Health & Place 33: 118–124. 25817940 10.1016/j.healthplace.2015.02.011

[bibr23-03091325241269757] Gaetz S Dej E Richter T (2016) Homelessness Canada in the state of 2016. Available at: https://www.deslibris.ca/ID/10065873.

[bibr24-03091325241269757] Gesler WM (1992) Therapeutic landscapes: medical issues in light of the new cultural geography. Social Science & Medicine 34(7): 735–746. 1376497 10.1016/0277-9536(92)90360-3

[bibr25-03091325241269757] Gowan T (2010) Hobos, Hustlers, and Backsliders: Homeless in San Francisco. Minneapolis: U of Minnesota Press.

[bibr26-03091325241269757] Herman JL (1992) Complex PTSD: a syndrome in survivors of prolonged and repeated trauma. Journal of Traumatic Stress 5(3): 377–391.

[bibr27-03091325241269757] Herman JL (2023) Truth and Repair: How Trauma Survivors Envision Justice. 1st edition. New York: Basic Books.

[bibr28-03091325241269757] Hopper EK Bassuk EL Olivet J (2010) Shelter from the storm: trauma-informed care in homelessness services settings. The Open Health Services and Policy Journal 3(2): 80–100.

[bibr29-03091325241269757] Hwang SW (2001) Homelessness and health. Canadian Medical Association Journal 164(2): 229–233. 11332321 PMC80688

[bibr30-03091325241269757] Jayne M Holloway SL Valentine G (2006) Drunk and disorderly: alcohol, urban life and public space. Progress in Human Geography 30(4): 451–468.

[bibr31-03091325241269757] Jayne M Valentine G Holloway SL (2008) Geographies of alcohol, drinking and drunkenness: a review of progress. Progress in Human Geography 32(2): 247–263.

[bibr32-03091325241269757] Johnsen S Cloke P May J (2005) Transitory spaces of care: serving homeless people on the street. Health & Place 11(4): 323–336. 15886141 10.1016/j.healthplace.2004.03.002

[bibr33-03091325241269757] Kearns RA Smith CJ (1994) Housing, homelessness, and mental health: mapping an agenda for geographical inquiry. The Professional Geographer 46(4): 418–424.

[bibr34-03091325241269757] Kerman N Kidd SA Voronov J , et al. (2023) Victimization, safety, and overdose in homeless shelters: a systematic review and narrative synthesis. Health & Place 83: 103092. 37515964 10.1016/j.healthplace.2023.103092

[bibr35-03091325241269757] Kneale J French S (2008) Mapping alcohol: health, policy and the geographies of problem drinking in Britain. Drugs: Education, Prevention & Policy 15(3): 233–249.

[bibr36-03091325241269757] Knowles C (2000) Burger King, Dunkin Donuts and community mental health care. Health & Place 6(3): 213–224. 10936776 10.1016/s1353-8292(00)00024-1

[bibr37-03091325241269757] Kramer TL Sigel BA Conners-Burrow N , et al. (2015) It takes a state: best practices for children exposed to trauma. Best Practices in Mental Health 11(1): 14–24.

[bibr38-03091325241269757] Krusi A Fast D Small W , et al. (2010) Social and structural barriers to housing among street-involved youth who use illicit drugs. In: Health & Social Care in the Community. Commerce Place, 350 Main St, Malden 02148, Ma Usa. Hoboken: Wiley-Blackwell. 10.1111/j.1365-2524.2009.00901.x PMC2883636 20102394

[bibr39-03091325241269757] Liddicoat S (2019) Affective spectrality in therapeutic space. Emotion, Space and Society 32: 100588.

[bibr40-03091325241269757] Linklater R (2020) Decolonizing Trauma Work: Indigenous Stories and Strategies. Nova Scotia: Fernwood Publishing.

[bibr41-03091325241269757] Lucero N Nutton J Scott K (2023) Beyond individual experience: frameworks for understanding shared group traumatization and healing. Montreal. Quebec.

[bibr42-03091325241269757] Malins P Fitzgerald JL Threadgold T (2006) Spatial ‘Folds’: the entwining of bodies, risks and city spaces for women injecting drug users in Melbourne’s Central Business District. Gender, Place & Culture 13(5): 509–527.

[bibr43-03091325241269757] Maté G (2008) In the Realm of Hungry Ghosts: Close Encounters with Addiction. New York City: Random House Digital, Inc.

[bibr44-03091325241269757] Mee K (2009) A space to care, a space of care: public housing, belonging, and care in inner newcastle, Australia. Environment & Planning A 41: 842–858. DOI: 10.1068/a40197.

[bibr45-03091325241269757] Miewald C McCann E McIntosh A , et al. (2018) Food as harm reduction: barriers, strategies, and opportunities at the intersection of nutrition and drug-related harm. In: Critical Public Health. 2-4 Park Square, Milton Park, Abingdon Ox14 4rn. Oxon, England: Taylor & Francis Ltd.

[bibr46-03091325241269757] Milligan C (2003) Location or dis-location? Towards a conceptualization of people and place in the care-giving experience. Social & Cultural Geography 4(4): 455–470.

[bibr47-03091325241269757] Milligan C (2014) Geographies of care. In: Cockerham WC Dingwall R Quah S (eds) The Wiley Blackwell Encyclopedia of Health, Illness, Behavior, and Society. Chichester, UK: John Wiley & Sons, Ltd, 683–685. Available at: https://doi.wiley.com/10.1002/9781118410868.wbehibs053.

[bibr48-03091325241269757] Milligan C (2017) Geographies of Care: Space, Place and the Voluntary Sector: Space, Place and the Voluntary Sector. Milton Park: Routledge.

[bibr49-03091325241269757] North CS Eyrich-Garg KM Pollio DE , et al. (2010) A prospective study of substance use and housing stability in a homeless population. Social Psychiatry and Psychiatric Epidemiology 45: 1055–1062. 19816646 10.1007/s00127-009-0144-z

[bibr50-03091325241269757] Olivet J McGraw S Grandin M , et al. (2010) Staffing challenges and strategies for organizations serving individuals who have experienced chronic homelessness. The Journal of Behavioral Health Services & Research 37(2): 226–238. 20052621 10.1007/s11414-009-9201-3

[bibr51-03091325241269757] Padgett DK (2007) There’s no place like (a) home: ontological security among persons with serious mental illness in the United States. Social Science & Medicine 64(9): 1925–1936. 17355900 10.1016/j.socscimed.2007.02.011 PMC1934341

[bibr52-03091325241269757] Padgett D Henwood BF Tsemberis SJ (2016) Housing First: Ending Homelessness, Transforming Systems, and Changing Lives. USA: Oxford University Press. 10.1176/appi.ps.671101 27903173

[bibr53-03091325241269757] Pain R (2019) Chronic urban trauma: the slow violence of housing dispossession. Urban Studies 56(2): 385–400.

[bibr54-03091325241269757] Pain R (2021) Geotrauma: violence, place and repossession. Progress in Human Geography 45(5): 972–989.

[bibr55-03091325241269757] Parr S (2019) The changing shape of provision for rough sleepers: from conditionality to care. Housing Studies 37: 1–24.

[bibr56-03091325241269757] Parsell C (2023) Homelessness: A Critical Introduction. Hoboken: John Wiley & Sons.

[bibr57-03091325241269757] Pauly B (2008) Harm reduction through a social justice lens. International Journal of Drug Policy 19(1): 4–10. 18226520 10.1016/j.drugpo.2007.11.005

[bibr58-03091325241269757] Power ER Williams MJ (2020) Cities of care: a platform for urban geographical care research. Geography Compass 14(1): e12474.

[bibr59-03091325241269757] Proudfoot J (2017) Drugs, addiction, and the social bond. Geography Compass 11(7): e12320.

[bibr60-03091325241269757] Proudfoot J (2019) Traumatic landscapes: two geographies of addiction. Social Science & Medicine 228: 194–201. 30925393 10.1016/j.socscimed.2019.03.020

[bibr61-03091325241269757] Reeves E (2015) A synthesis of the literature on trauma-informed care. Issues in Mental Health Nursing 36(9): 698–709. 26440873 10.3109/01612840.2015.1025319

[bibr62-03091325241269757] Rhodes T (2002) The ‘risk environment’: a framework for understanding and reducing drug-related harm. International Journal of Drug Policy 13(2): 85–94.

[bibr63-03091325241269757] Shaw IG (2019) Worlding austerity: the spatial violence of poverty. Environment and Planning D: Society and Space 37(6): 971–989.

[bibr64-03091325241269757] Shinn M Cohen R (2019) Homelessness prevention: a review of the literature. Center for Evidence-Based Solutions to Homelessness. https://www.evidenceonhomelessness.com/wp.

[bibr65-03091325241269757] Tyner JA (2020) The slow and the fast violence of displacement. In: Adey P Bowstead JC Brickell K , et al. (eds) The Handbook of Displacement. Cham: Springer International Publishing, 79–88. DOI: 10.1007/978-3-030-47178-1_5.

[bibr66-03091325241269757] Victoria Cool Aid Society (2024) Victoria Cool Aid society. Available at: https://coolaid.org/.

[bibr67-03091325241269757] Willse C (2015) The Value of Homelessness: Managing Surplus Life in the United States. Minneapolis: U of Minnesota Press.

[bibr68-03091325241269757] Wilton R Moreno CM (2012) Critical geographies of drugs and alcohol. Social & Cultural Geography 13(2): 99–108.

